# Psychiatric nurses’ experiences after the closure of Life Esidimeni psychiatric care centres

**DOI:** 10.4102/curationis.v45i1.2332

**Published:** 2022-12-02

**Authors:** Annie Temane, Maide Manamela, Marie Poggenpoel

**Affiliations:** 1 Department of Nursing, Faculty of Health Sciences, University of Johannesburg, Johannesburg, South Africa

**Keywords:** psychiatric nurses, closure, mental health care users, experience, Life Esidimeni care centres

## Abstract

**Background:**

A national tragedy occurred between October 2015 and June 2016 when psychiatric patients with profound intellectual disabilities were transferred from psychiatric care centres to non-governmental organisations (NGOs). The process of transferring psychiatric patients had severe consequences for psychiatric patients and psychiatric nurses.

**Objectives:**

The study’s objective was to explore and describe psychiatric nurses’ experiences after the closure of Life Esidimeni psychiatric care centres.

**Method:**

A qualitative, exploratory, descriptive and contextual research design was employed. Semi-structured interviews were utilised to collect data. Purposive sampling was utilised to select eight psychiatric nurses to participate in the study. Data were analysed using Tesch’s thematic method of coding.

**Results:**

The analysis of data revealed the following themes: With the closure of the care centres participants experienced (1) shock, dismay and life interruption; (2) trauma related to the disintegration, of psychiatric patients’ lives, their own families and work-life and (3) sense of resilience.

**Conclusion:**

From the findings, it is clear that the psychiatric nurses needed support as evidenced by the challenges they experienced. The healthcare professionals in mental health and mental health nursing post-graduate students could conduct further research focusing on the experiences and the impact that the closure of Life Esidimeni psychiatric care centres have on the psychiatric nurses’ mental health.

**Contribution:**

This study contributes to the body of knowledge in psychiatric nursing by highlighting the impact of hospital closure on psychiatric nurses.

## Introduction

Mental health services in South Africa have been in the spotlight since October 2015, when the Member of Executive Council (MEC) of Health in Gauteng decided to end a 40-year contract between the Department of Health and Life Esidimeni for the provision of mental health services around Gauteng province, South Africa. The Life Esidimeni psychiatric care centres were among the community-based service facilities offering care to chronic psychiatric patients who had no family support or the means to receive private care. The seven centre-based service facilities offered chronic mental health care, frail care and substance abuse recovery. Durojaye and Agaba (2018:161) reported that between October 2015 and June 2016, 1711 psychiatric patients were relocated from mental health facilities operated by Life Esidimeni psychiatric care centres in Gauteng province to alternate facilities managed by multiple non-governmental organisations (NGOs). The transfers of psychiatric patients from their known mental health care establishment, Life Esidimeni to unknown mental health care establishments resulted in the deaths of 144 psychiatric patients. Furthermore, with these transfers of psychiatric patients, the Department of Health was unable to ascertain the whereabouts of an additional 44 psychiatric patients (Freeman 2018:1).

According to Child (2017:16), when the MEC of Health in Gauteng province announced the termination of the subsidised contract with Life Esidimeni psychiatric care centres, the NGO facilities to which psychiatric patients were transferred were ill prepared and ill equipped for the influx of psychiatric patients. Furthermore, the disaster drew public attention in September 2016, when the Executive Council for Health members informed parliament that approximately 36 former residents of Life Esidimeni had died under mysterious circumstances following their transfers to the NGOs (Durojaye & Agabe 2018:161). According to Durojaye and Agaba (2018:161), the Minister of Health in South Africa requested that the newly established office of the Health Ombudsman investigates the circumstances surrounding the deaths of psychiatric patients and advises on the way forward.

### Problem statement

The Life Esidimeni tragedy was in the media and news from March 2016 (Makgoba 2017:1) when the Health Ombudsman convened an arbitration to seek justice for the affected psychiatric patients and their relatives. According to the report titled ‘The Circumstances Surrounding the Deaths of Mentally Ill Patients’ (Makgoba 2017:55), consideration was given to the psychiatric patients and their relatives; however, Durojaye and Agaba (2018:162) indicate that no report was presented on how psychiatric nurses were considered or affected by the closure of Life Esidimeni psychiatric care centres. Although mental health care users (MHCUs), their families and the NGOs where MHCUs were transferred were profiled many times in the news and media, there was a lack of consideration of how the psychiatric nurses who were working at these centres were coping. A psychiatric nurse in a South African context is a nurse with a basic nursing qualification and an additional qualification in psychiatric nursing sciences. In this study, psychiatric nurses refer to nurses who worked at Life Esidimeni psychiatric care centres.

There was a lack of communication to psychiatric nurses about the Marathon Project of transferring MHCUs to unlicensed NGOs and about the closure of the Life Esidemini centres. There is a need to understand the psychiatric nurses’ experiences regarding the closure of Life Esidimeni psychiatric care centres. The study sought to explore and describe the experiences of psychiatric nurses after the closure of Life Esidimeni psychiatric care centres in Gauteng province, South Africa.

### Aim of this article

The aim of this article is to share the findings on the psychiatric nurses’ experiences after the closure of Life Esidimeni psychiatric care centres in Gauteng province, South Africa.

## Research method and design

A qualitative (Holloway & Galvin 2017:3), exploratory, descriptive (Gray, Grove & Sutherland 2017:76) and contextual research design (Holloway & Galvin 2017:4–5) was employed. The qualitative research approach allowed the researcher to emphasise the dynamic, holistic and individual aspects of the psychiatric nurses’ experiences after the closure of Life Esidimeni psychiatric care centres in Gauteng province, South Africa. An exploratory research design uncovered the experiences of psychiatric nurses. A descriptive approach was followed to describe the psychiatric nurses’ experiences as accurately as possible. The contextual research approach ensured sensitivity to the research context; in this study, the context was the homes of the psychiatric nurses who worked at Life Esidimeni psychiatric care centres in Gauteng province, South Africa. The collected data were contextualised into current literature on the closure of Life Esidimeni psychiatric care centres in Gauteng province, South Africa.

### Research setting

The study took place at the homes of the psychiatric nurses who worked at Life Esidimeni psychiatric care centres at the time of closure.

### Population and sample

The population was psychiatric nurses who were employed at Life Esidimeni psychiatric care centres at the time of their closure. The research sample of eight participants was drawn from the population of 13 psychiatric nurses who worked at Life Esidimeni psychiatric care centres during their closure. A purposive sampling method was used to select psychiatric nurses. In qualitative research, the purposeful sampling approach is used to shed light on a new field of inquiry or to achieve a comprehensive knowledge of a complex experience or event (Gray et al. 2017:539). The sample size was determined by data saturation, which occurred when no new ideas or information were shared by the participants (Gray et al. 2017:352); this occurred at the eighth interview. As a result, the researcher used saturation to justify the sample size and number of participants in this study.

The participants met the following inclusion criteria:

- Psychiatric nurses employed at the Life Esidimeni psychiatric care centres in Gauteng province at the time of their closure.
- Nurses transferred to work at a psychiatric hospital in Gauteng province after the closure of Life Esidimeni psychiatric care centres.
- Psychiatric nurses with 2 to 5 years’ experience working at Life Esidimeni psychiatric care centres in Gauteng province.
- Willingness to participate in the study and share experiences about the closure of the Life Esidimeni psychiatric care centres research phenomenon.

In this study, the exclusion criteria were other categories of nurses, namely auxiliary nurses, enrolled nurses and nurse managers of Life Esidimeni’s closure in Gauteng province, South Africa who were not accessible population for this part of the study.

### Data collection procedure

Permission was sought from the hospital’s Chief Executive Officer and the operational managers where the psychiatric nurses were transferred. The recruitment took place from March to May 2020. Data were collected by conducting individual, semi-structured interviews with participants who met the inclusion criteria. The date, time and place to conduct interviews were confirmed by the participants. The interviews were conducted between June 2020 and September 2020. The participants provided a quiet area in their homes where there were no interruptions by other family members during the interview process. Data collection was done over four months. Each interview session lasted approximately 45–60 min, was audio recorded with the participants’ permission and transcribed before being analysed. During the interviews, the researcher took field notes and asked probing questions to ensure a detailed exploration of participants’ experiences. The interviews were facilitated using open-ended questions, and facilitative communication skills were used to obtain in-depth information from the participants.

Field notes and observational notes were made during the data collection procedure. Data were then analysed according to Tesch’s method of open coding (Creswell & Poth 2018:185–189). Data saturation was achieved when neither new themes nor new data emerged, and this occurred in the eighth interview. This study was a narrow scope of focus on the psychiatric nurses’ experiences after the closure of Life Esidimeni Psychiatric Care Centres. According to Gray et al. (2017:550), a study with a broad scope requires more sampling of participants, events or documents than a study with a narrow scope.

### Data analysis

Data analysis using Tesch’s thematic coding method continued concurrently with data collection because the researcher reflected on the raw data as these became available. All audio-recorded interviews were transcribed verbatim. During the first analysis step, all the transcripts of psychiatric nurses’ experiences were read and ideas were jotted down as they came to mind. The second step involved, making notes in the margins about the ideas and the meanings of the content of the transcripts to understand the underlying meaning of the information that emerged through the participant’s words. In step three, a list of topics and those with similar meaning were clustered together. Topics were formed into columns arranged as major topics, unique topics and leftovers. The transcripts were thus individually analysed to establish key emerging themes. In the fourth step, the researcher abbreviated the topics as they were made into codes and were written next to the appropriate text segments. In step five, the descriptive wording for the topics was arranged into categories. Similar topics were grouped together in order to reduce the list of categories. Themes, regularities and patterns in the data were examined and placed in a table format. During the sixth step, key themes were derived from narrative interviews. For step seven, the material belonging to each category were assembled and a preliminary analysis was performed.

During coding, the data were broken down into small parts, and the parts were closely examined for similarities and differences. Themes were identified and analysed, and then an independent coder confirmed the formulated codes. The independent coder holds a doctorate in Psychiatric Nursing Science and has extensive experience in qualitative data analysis. A consensus discussion was scheduled with the independent coder to reach a consensus on the identified themes and categories to determine the accuracy of the transcribed data’s interpretation.

### Ethical considerations

The Research Ethics Committee at the University of Johannesburg (REC-709-2020), Higher Degrees Committee (HDC-01-72-2020), National Health Research Database (GP_202010_05) and Weskoppies Psychiatric Hospital approved this study.

The following four fundamental ethical principles were applied while conducting the study: autonomy, non-maleficence, beneficence and justice. The researcher ensured the principle of autonomy by respecting the participants’ right to anonymity and confidentiality. Thus, the participants’ real names were not used in the study; instead, participants were referred to by a number. In addition, collected data were only accessible to the researcher, independent coder and the two research supervisors. The principle of non-maleficence and beneficence by ensuring the participants were comfortable by asking appropriate questions to avoid discomfort and ensured participants’ emotional support; the psychologist was available in the case of any emotional support needed. In order to ensure the principle of justice, participants were informed that participation in the study was voluntary, and withdrawal from the study was welcomed, without any penalties.

### Trustworthiness

Lincoln and Guba’s framework was used to ensure trustworthiness (Polit & Beck 2017:768). The following measures to ensure trustworthiness were adhered to throughout the study: credibility, transferability, dependability and confirmability.

Credibility was maintained through member checking and data triangulation. In this study, data triangulation was ensured by using different data collection methods, such as individual, in-depth, interviews with all participants, observations and field notes. The researcher used semi-structured interviews and field notes to ensure data confirmation. A dense description of the participants’ demographics and rich descriptions of the findings – with direct verbatim quotations from the interviews – were provided to ensure presentation of credible data. Purposive sampling further promoted transferability since the researcher purposefully selected participants with knowledge of the research topic.

Dependability, which relates to the consistency of the findings, is when a study has to provide the readers with evidence that if redone with the same participants in a similar context its finding would be repeated (Korstjens & Moser 2018:121). The interview transcripts, coding material and field notes were kept as audit trail throughout the study. The dense description of the research methodology was presented.

Confirmability was ensured through ongoing analysis and member checking. The researcher ensured member checking by meeting with the participants individually at the end of data collection and analysis. The researcher asked participants to review and comment on the data collection. This was done to confirm and validate what the participants shared during the data collection phase.

## Findings

### Description of the participants

Eight psychiatric nurses who worked at Life Esidimeni psychiatric care centres at the time of closure were interviewed after they all gave consent to participate in the study. They were between 32 and 45 years old, seven were female, and one was a male psychiatric nurse. Participants were parents and breadwinners in their families. Participants were from different provinces in South Africa; four participants were originally from Limpopo province, one was from KwaZulu-Natal province and three participants were originally from Gauteng province but had settled around Gauteng province to be close to their place of employment. The demographic findings revealed that while employed at Life Esidimeni care psychiatric centres, the participants worked near their residence, which they shared with their children, spouses and other family members.

Participants had to relocate to the psychiatric hospital in Pretoria where some of the MHCUs were transferred. Participants were interviewed in English, and data saturation was reached when no new themes emerged, and this occurred in the eighth interview. The participants’ demographics are presented in Table 1.

**TABLE 1:** Participants’ demographics.

Participant numbers	Age (years)	Gender	Race	Years of employment at Life Esidimeni psychiatric care centre (years)
1	35	Female	African	10
2	40	Female	African	15
3	45	Female	African	15
4	32	Female	African	6
5	45	Female	African	12
6	38	Male	African	13
7	35	Female	African	8
8	40	Female	African	15

*Source*: Adapted from Manamela, M., Temane, A. & Poggenpoel, M., 2022. ‘“Psychiatric nurses” lived experiences after the closure of Life Esidimeni psychiatric care centres’, Masters thesis, University of Johannesburg, Johannesburg, viewed n.d., from https://hdl.handle.net/10210/499007

### Presentation of findings

The three themes that emerged from the data were: (1) shock, dismay and life interruption; (2) trauma related to the disintegration of psychiatric patients’ lives, their own families and work-life and (3) sense of resilience. These themes and sub-themes are discussed in the sections that follow.

Perhaps make a Table 2 here and present the themes and subthemes.

**TABLE 2:** Themes and Sub-themes of psychiatric nurses’ experience after the closure of Life Esidimeni psychiatric care centres

Themes	Sub- themes
Shock, dismay, and life interruption	Psychiatric nurses experienced emotional turmoil due to the unexpected closure of the care centres
	Impact on personal and professional roles
	Financial impact
	Work-life turmoil
Trauma related to the disintegration of psychiatric patients’ lives, their own families, and their work-life	Traumatic experiences witnessing the unfair treatment of vulnerable psychiatric patients
	Work disintegration of psychiatric nurses
Sense of resilience	Psychiatric nurses had a positive outlook on starting new relationships

### Theme 1: Shock, dismay and life interruption

This theme reflects the psychiatric nurses’ experiences with the care centres’ closure. The four sub-themes that emerged from this theme are (1) psychiatric nurses experienced emotional turmoil because of the unexpected closure of the care centres; (2) negative impact on personal and professional roles; (3) financial impact and (4) work-life turmoil.

#### Sub-theme 1.1: Psychiatric nurses experienced emotional turmoil because of the unexpected closure of the care centres

The participants mentioned that the speed and scale of psychiatric patients’ transfers to different NGOs and public hospitals in Gauteng province, and the way the psychiatric patients were transferred (some in transport that was inappropriate for this purpose), resulted in many psychiatric patients losing their lives. It was a painful, sad and traumatic experience. They further indicated that the closure of Life Esidimeni psychiatric care centres was sudden, and they had short notice to find new employment. The nurses were terminated but ultimately transferred to other institutions for work. In addition, participants were immensely sad to leave their families behind when they were transferred to work in a different area.

One of the participants mentioned she was preparing for her wedding when the unexpected closure occurred. She said:

‘The closure came as a shock to me because at the time I was staying in Randfontein with my son and my fiancé, which was convenient for us because, for his school, my workplace and my fiancé’s workplace, now because of the sudden closure, I had to move to Pretoria, this closure messed up my life … Worst of all my fiancé and I were busy planning for our wedding at the time the closure occurred, as I’m sitting here with you, my relationship with the father of my child does no longer exist, because he could not bear the fact that I have to relocate to Pretoria.’ (Participant 3, female, 40 years old, 15 years of employment at Life Esidimeni)

Another participant said:

‘we, as the staff of Life Esidimeni Care Centres, were given notice letters that the Care Centres are closing and I was one of the Psychiatric nurses who was placed at Weskoppies hospital, and I have to report there on the 1st of June 2016, so, to me it came as a shock, because it was short notice, I didn’t even know where Weskoppies was in Pretoria, I have never been there as I was staying in Vosloorus in the East Rand working at Waverly Care Centre, then suddenly “boom” go to Weskoppies’ It was shocking, Sister … to think that we started by hearing rumours that the Care Centres will close down … And I feel that the leaders of Life Head office could have alerted us some time before the actual closure regarding the movements so that we can plan accordingly.’ (Participant 1, female, 35 years old, 10 years of employment at Life Esidimeni)

During the interviews, participants expressed dissatisfaction about the speed with which the closure of Life Esidimeni psychiatric care centres in Gauteng province occurred. They were also not happy about the rate with which psychiatric patients were moved, as they felt they were not given enough time to prepare for the move. There were no prior discussions about the possible closure and their transition to different public hospitals around Gauteng province. Participants thus felt betrayed by their previous employer, who left them unprepared. The psychiatric nurses who participated in this study shared unique stories about their experiences as a result of the care centres’ closure. One participant said:

‘I think that at least the leadership of Life Health office should have alerted the employees prior to the movement and closure, maybe at least a year in advance, so that we could make plans of finding new employment to prevent frustrations that people like myself had to experience.’ (Participant 1, female, 35 years old, 10 years of employment at Life Esidimeni)

She continued:

‘The hospital managers were the only ones attending the meetings regarding the closure, “so I feel we were not given a chance to choose where we want to go”.’ (Participant 1, female, 35 years old, 10 years of employment at Life Esidimeni)

#### Sub-theme 1.2: Impact on personal and professional roles

In this study, the participants were parents, guardians and a source of support in their families. During the interviews, participants mentioned feeling deprived of their fulfilling roles in their families and work because of the care centres’ closure:

‘My experiences of the closure are very sad, indeed, frustrating. I wish the closure never happened in the first place, because most of us were negatively affected in our lives, and our families.’ (Participant 6, male, 38 years old, 13 years of employment at Life Esidimeni)

The participants who had to leave their young children at home when they relocated shared that they felt motherhood had been taken from them in a significant way. One of the participants explained:

‘I feel sad, I feel like the closure did not happen, and I could not have left my kids alone with their father, especially the girl child, I feel like I’m failing as a parent.’ (Participant 5, female, 45 years old, 12 years of employment at Life Esidimeni)

Participants experienced family disruption because of leaving their family behind and having to find accommodation closer to the new place of work. One participant said:

‘I feel that the closure could not have happened because it was too sudden and just caused a disruption in our families, now I have to stay far from my family, as I’m the sole provider at home, it’s very difficult.’ (Participant 2, female, 40 years old, 15 years of employment at Life Esidimeni)

#### Sub-theme 1.3: Financial impact

The participants reported experiencing financial constraints and shared that they experienced difficulties making ends meet because of their relocation. They verbalised that cost of living was challenging, especially travelling costs for those travelling from Randfontein to Pretoria to get to work, as they could find no alternative, affordable accommodation in the area. Participants said:

‘The closure was very sudden, and most of us were not emotionally and financially prepared to be moved to new hospitals, and we were not given time to prepare ourselves, and she further said, “The closure of Life Esidimeni was very traumatic one for me and I was stressed indeed”.’ (Participant 5, female, 45 years old, 12 years of employment at Life Esidimeni)

‘Yes, now the problem is that I have to travel to and from work, this affects me financially.’ (Participant 6, male, 38 years old, 13 years of employment at Life Esidimeni)

#### Sub-theme 1.4: Work-life turmoil

The participants narrated there was no support from management when they complained about their colleagues’ unfair treatment. A participant relayed:

‘I just ignored them because knowing how to control my anger, is the best solution for me, I just continue working as if nothing is happening, the problem is even if I can report to the Operational manager to raise my concerns about the name-calling, little was done, because here the staff always run to the union when they are being corrected, “yah … what can I do, at least I still have a job”.’ (Participant 8, female, 40 years old, 15 years of employment at Life Esidimeni)

The participants continued to share how they endured rejection and verbal abuse in their new place of employment. Some were also falsely accused of causing some of the Esidimeni psychiatric patients’ deaths. A participant mentioned:

‘Some of the staff members did not receive me and my other colleagues from Life Esidimeni very well, as I could sense a lot of rejection from what they were saying about Life Esidimeni, saying how did we get the jobs without being interview as their children are struggling to find jobs, and there was no proper orientation.’ (Participant 7, female, 35 years old, 8 years of employment at Life Esidimeni)

### Theme 2: Trauma related to the disintegration of psychiatric patients’ lives, their own families and their work-life

This theme reflects the tragic disintegration participants experienced. The two sub-themes that emerged from this theme are: (1) traumatic experiences witnessing the unfair treatment of vulnerable psychiatric patients and (2) work disintegration of psychiatric nurses.

#### Sub-theme 2.1: Traumatic experiences witnessing the unfair treatment of vulnerable psychiatric patients

The participants in this study shared their traumatic experiences witnessing the unfair treatment of vulnerable psychiatric patients during the closure of Life Esidimeni psychiatric care centres. One participant said:

‘It was a very traumatic experience for me to see the chronically ill psychiatric patients being moved so quickly to be taken to some NGOs, and some went to Sterkfontein and others to Weskoppies hospital, having to watch helplessly when physically disabled psychiatric patients being carried in bakkies …’ (Participant 1, female, 35 years old, 10 years of employment at Life Esidimeni)

#### Sub-theme 2.2: Work disintegration of psychiatric nurses

The psychiatric nurses who worked at Life Esidimeni psychiatric care centres at the time of the closure shared that they were being treated unfairly at their newly relocated workplace. A lack of support networks that were established at their previous workplace, a lack of support from their employer in terms of relocation and not being financially compensated were additional challenges.

The psychiatric nurses said they faced unfair treatment in their new jobs and their efforts of reporting these incidents were in vain. Participants stated they had to endure these challenges. One of the participants said:

‘Some of the staff in my new job called us names, like Esidimeni … or absorption, each time in the ward there was a mistake, the blame will be put towards us the staff from Esidimeni.’ (Participant 3, female, 40 years old, 15 years of employment at Life Esidimeni)

She continued:

‘At times I just don’t feel like waking up and going to work because of these challenges.’ (Participant 3, female, 40 years old, 15 years of employment at Life Esidimeni)

The participants stated that they left all their support networks behind to keep their job. One of the participants who was busy preparing for her wedding at the time of the closure mentioned:

‘The sad thing is that I was preparing for my wedding at that time, now, my fiancé and I, are no longer together because he could not stand the fact that I was going to relocate to Pretoria so suddenly.’ (Participant 3, female, 40 years old, 15 years of employment at Life Esidimeni)

The participants experienced that the investigation surrounding the care centres’ closure focused mainly on the psychiatric patients’ families who received financial compensation, while no monetary aid was allocated to staff. A participant reported:

‘My concern is that the government only concentrated on investigating about the psychiatric patients and their families, but they never thought about how the sudden closure impacted our lives as the previous staff of Esidimeni.’ (Participant 7, female, 35 years old, 8 years of employment at Life Esidimeni)

The psychiatric nurses struggled to focus on their jobs as they were far from their families but still had to manage challenges at home. Consequently, some participants felt unable to cope in their new workplace. Participants explained they are always thinking of home when they are at work and are not coping. They said:

‘I used to cry a lot when I talked to my mother over the phone, I cannot say exactly I’m coping, it is really difficult for me, my elderly mother is sickly and has to look after my daughter who is Asthmatic [*with tears in her eyes*] some of the days, I have to rush home, leaving my work and this affect my work performance at work.’ (Participant 4, female, 32 years old, 6 years of employment)

### Theme 3: Sense of resilience

This theme reflects the participants’ responses when they were asked how they were rebuilding their lives. One sub-theme emerged from this theme: (1) Psychiatric nurses had a positive outlook on starting new relationships, which is discussed next.

#### Sub-theme: 3.1 Psychiatric nurses had a positive outlook on starting new relationships

The participants stated they would like to be transferred to public hospitals nearer home. Therefore, though they faced challenges in getting new posts or cross-transfers, they continuously approached human resources (HR) with transfer requests. One of the participants said:

‘I’m planning to keep looking for a job in the East Rand hospitals, as I’m from that side, though I tried without any luck, I will continue to check with the Human resources manager if there are any cross transfers.’ (Participant 1, female, 35 years old, 10 years of employment at Life Esidimeni)

Despite their circumstances, the psychiatric nurses were grateful for support from new friends, families and previous colleagues from Esidimeni. Support is important for psychiatric nurses since the literature suggests social support facilitates adjustments within new environments (Mendieta 2019:110).

One participant said:

‘I had to ask my cousin to look after my son until I sorted getting a new school for him in Pretoria, because I could not just remove him from his previous school without proper arrangements, at least she was very supportive, as I was busy looking for accommodation in Pretoria.’ (Participant 3, female, 40 years old, 15 years of employment at Life Esidimeni)

## Discussion of findings

This study addressed the experiences of psychiatric nurses after the closure of Life Esidimeni psychiatric care centres. The results revealed that the psychiatric nurses had negative experiences after the closure of Life Esidimeni psychiatric care centres. Brame (2017:16) states that the closure of a hospital generates job losses. Some employees may find alternative employment within the community, while others, especially health professionals, often have to leave their community to find employment in other areas. This causes financial stress, and it becomes difficult for the dependents left behind by the person relocating to other areas for employment.

The closure of the care centres caused mental health challenges such as anxiety and emotional distress for the psychiatric nurses because of the relocation to another workplace. According to Brooks (2019:4), uncertainty only fuels the flames of anxiety, and new workplace anxiety often relates to the upcoming changes. These changes can be experienced as traumatic. According to Kendall (2018:20), trauma is an emotional and psychological experience typically attributed to an extraordinarily stressful event that shatters one’s sense of security, making one feel helpless in a dangerous world. Psychological trauma can leave one struggling with emotions, memories and anxiety that persist. Traumatic experiences can cause social isolation and loneliness and can negatively impact one’s mental health that is emotional, psychological and social aspects of one’s life.

Wu (2020:27) states that social isolation and loneliness are major risk factors that have been linked to poor mental health status. Hartmaan (2017:16) concurs that social isolation and loneliness can result in the loss of relationships and support systems. However, Fanari and Segrin (2021:298) contradict these findings stating that although feelings of sadness, frustration, loneliness and withdrawal may occur from cultural and interpersonal environments; however, integration into aspects of the new environment and home culture may lead to a successful readjustment.

Changing working environments is inevitable in organisations, and when it happens, leadership often underestimates the impact those changes have on employees. Brooks (2019:54) states that a workplace is typically an environment where people have different personalities and communication styles. Different sources of workplace issues can also ultimately lead to stress, discrimination and unfair treatment among employees. This was reflected in this study as participants reported on their negative experiences with the staff in their new work environment. This is supported by Teo et al. (2021:10) who found that workplace bullying exposes employees to psychological stress; furthermore, they found that employees would react emotionally to the negative affective events that would affect their subsequent behaviours, attitudes and well-being in responding to the respective event. Participants further indicated they were not given a choice in terms of where they would prefer to work after the unions negotiated for them to be absorbed by government hospitals; hence, most had to settle for jobs far from their homes.

The participants experienced a lack of support from their employer when they could not get accommodation and participants further shared how they struggled during the relocation. The participants reportedly tried several avenues of approaching the HR department to let them know when there are vacancies in public hospitals nearer their homes, to be considered for possible transfer. However, despite the attempts by several participants to get HR managers’ help, little was done and participants were unsuccessful in their requests. According to Morris (2021:1), relocation help occurs when a corporation or institution assists new or present employees in transferring for work from one location to another. This is not possible in the South African context as government employees are not assisted with relocation. In this study, the participants stated they were left to make their own arrangements to find suitable transport to their new place of work.

The participants expressed they had left all their support networks behind to remain employed and not lose their income. In addition, they did not even know the areas to which they were relocated and had no friends or relatives nearby. The importance of family to promote emotional well-being cannot be underestimated. Devlin et al (2019:320) agrees that family members are the people you surround yourself with the most, who give your life meaning and for whom you would do anything.

In addition, the study found family strain was caused by the absence of a parent or spouse from the household. Seepamore (2021:45) suggests that the absence of one spouse from home places the burden of household chores and children on the shoulders of the partner left behind. Moreover, working-away employees often cannot wait to go home during leave or time off duty; hence, they are reported to cope poorly in an environment away from the family unit (Mendieta 2019:187).

This study found that participants were parents who had to leave their children in the care of either their spouses or elderly parents. They reported it was difficult to focus and cope in their new workplace. Participants explained they are always thinking of home, and wondering about their children; as a result, complaints and conflicts arose from their partners as they also struggled with the burden of raising children alone.

The findings further revealed that the psychiatric nurses who moved to different hospitals around Gauteng province, South Africa after the closure of Life Esidimeni psychiatric care centres showed resilience by making plans to better their own circumstances. Masten et al (2008:425) define resilience as the ability to successfully adjust and have positive functioning, or competence despite high-risk status, persistent stress or severe trauma. The participants started to rebuild a substitute support network and showed a positive outlook. Participants kept praying for a solution and were grateful that they still had jobs, despite the challenges they experienced. Participants bought homes and rented flats closer to their place of work and seemed to have reorganised their families’ lives so that everyone could be taken care of, while participants had joined recreational activities in the hospital for staff, such as the hospital choir, just to take their minds off the challenges they faced. Five years down the line, they still resiliently survived some lasting disruptions, making plans to rebuild their lives. Most came up with positive solutions to try and remedy some of their challenges, and the psychiatric nurses managed to cope better after building new relationships in their new workplace and getting continuous support from families and friends.

### Limitations

The first limitation of this study was that it focused only on a specific category of nurses. The second limitation was that even though data saturation was reached by the eighth interview, the sample size was a small sample; therefore findings can only be inferred to other contexts and not be generalised.

### Recommendations

Based on the study’s findings, the following recommendations are made:

- Psychiatric nurses who worked at Life Esidimeni psychiatric care centres should be included in the HR wellness programmes in order to promote their mental health, that is emotional, psychological and social well-being.
- Health care professionals in mental health should mobilise resources and implement platforms to teach psychiatric nurses how to apply measures that will promote their mental health.

Literature indicates that support programmes should also include skills for families, groups and individuals so that they can modify their attitudes and enhance positive coping mechanisms (Dickerson 2017:3–4).

## Conclusion

Health care professionals in mental health and post-graduate students could conduct further research focusing on the experiences and the impact that the closure of Life Esidimeni Life Esidimeni psychiatric care centres on their mental health. Mental health programmes such as debriefing, support groups and individual therapy could be implemented to provide support for the psychiatric nurses. This study provides literature on the events that affected the nurses because of the Life Esidimeni Care Centre closure.
